# Editorial: Understanding the role of gut hormones, microbiota, and miRNAs in metabolic regulation and glucose homeostasis in obesity and type-2-diabetes

**DOI:** 10.3389/fendo.2023.1255942

**Published:** 2023-08-18

**Authors:** Lisa M. Matz, Ramasatyaveni Geesala, Ravinder Reddy Gaddam, Xuan-Zheng Shi

**Affiliations:** ^1^ Center for Precision Environmental Health, Baylor College of Medicine, Houston, TX, United States; ^2^ Department of Internal Medicine, Division of Gastroenterology and Hepatology, The University of Texas Medical Branch, Galveston, TX, United States; ^3^ Department of Internal Medicine, Division of Cardiovascular Medicine, The University of Iowa, Iowa City, IA, United States

**Keywords:** gut hormones, microbiota, miRNAs, obesity and type 2 diabetes, glucose homeostasis, metabolism

Obesity and diabetes are growing global health concern, as the number of adults living with diabetes globally has more than quadrupled since 1980 (1). In the U.S., 11.3% of the population are living with type 2 diabetes, and many more with prediabetes (2). Impaired glucose homeostasis and metabolic dysregulation are key features of obesity and type 2 diabetes, and gut hormones, microbiota, and microRNAs are critical mediators of this process. Although genetics certainly has a role to play in this process, there is a growing recognition of the role that the environment and holobiont (host plus commensal microbes), and its associated hologenome, contribute to host physiology and disease outcomes (3–5). In this Research Topic, we explore the interplay between gut hormones, microRNAs, and microbiota in glucose homeostasis, and how perturbations to these systems lead to metabolic dysregulation.

Specifically, here, Farhat et al. review microbiome changes that occur in women with gestational diabetes mellitus and their offspring, while Liu S. et al. investigate the interaction of the microbiome and testosterone levels in men with type 2 diabetes. Although reproducibility in microbiome research is challenging due to the considerable variability in extraction, library preparation, sequencing, and analysis pipelines, the increasing application of meta-transcriptomics will aid in identifying functional convergences that will be of critical value in informing disease progression and treatment.

Additionally, Liu C. et al. discuss mechanisms of GLP-1-mediated regulation of glucose homeostasis, including circadian rhythm and its disruption. The microbiome is no exception to circadian rhythms, and Liu C. et al. explore some of these intersecting pathways and report on their impact on GLP-1 secretion. Finally, Palihaderu et al. provide insight into the role that microRNAs - RNAs that negatively regulate the target gene expression either by degradation or translational repression of targeted mRNAs – play in insulin resistance. Importantly, both microRNAs and gut hormones are implicated in abnormal glucose homeostasis in obesity and diabetes and are emerging as important targets to treat diabetes-associated complications.

We are optimistic that future directions of these research areas will include bench-to-bedside applications to target enteroendocrine factors or miRNAs to treat diabetes.

## Author contributions

RG: Writing – review & editing. LM: Writing – original draft. RRG: Writing – review & editing. XS: Writing – review & editing.
